# Supplementary material for: CLICs-dependent chloride efflux is an essential and proximal upstream event for NLRP3 inflammasome activation
Source: Nat Commun. 2017 Aug 4;8:202. doi: 10.1038/s41467-017-00227-x (PMC5544706; doi:10.1038/s41467-017-00227-x)
Supplement: Supplementary file 1 — Supplementary Information [file 41467_2017_227_MOESM1_ESM.pdf]

File name: Supplementary Information
Description: Supplementary figures and supplementary tables.

File name: Peer Review File
Description:

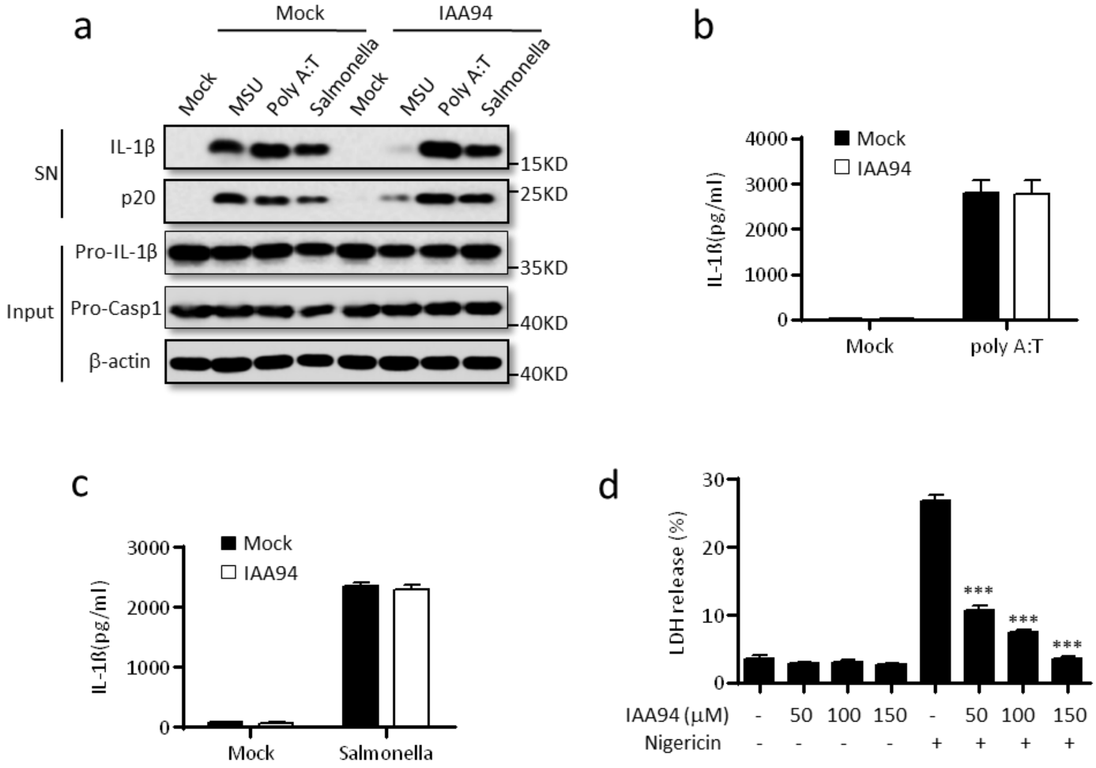

Supplementary Fig.1. IAA94 has no effect on AIM2 and NLRC4 inflammasome activation. (**a-c**) Immunoblot (**a**) or ELISA (**b, c**) analysis of IL-1β and cleaved caspase-1 (p20) in culture supernatants of LPS-primed BMDMs treated with IAA94 (150 μM) and then left stimulated with cytosolic poly A:T or salmonella. (**d**) Assay for LDH release in the culture supernatants of LPS-primed BMDMs treated with different doses of IAA94 and then left stimulated with nigericin. Two-way ANOVA. Data are from three independent experiments with biological duplicates in each (**b-d**; mean ± SEM of $n = 6$) or are representative of three independent experiments (**a**). *** P<0.001.

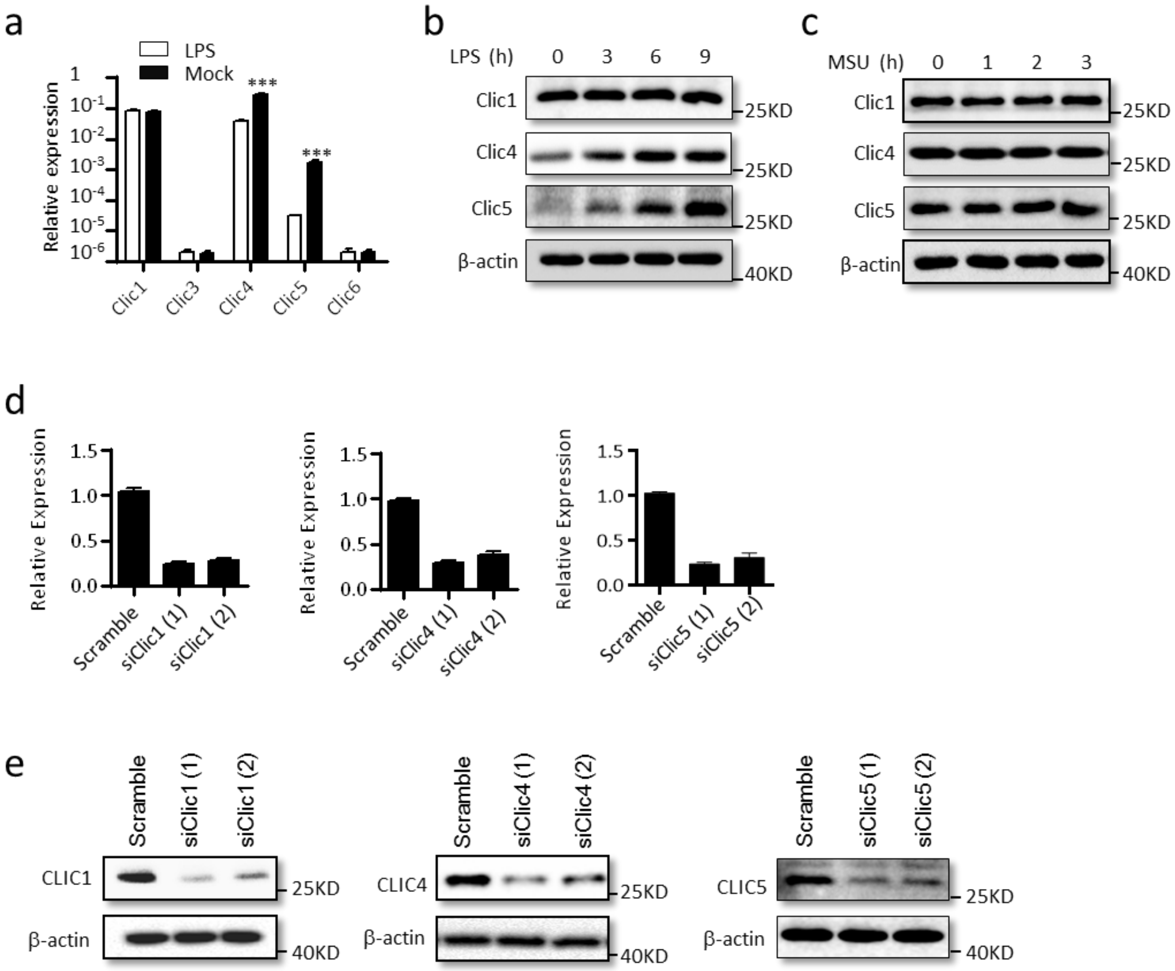

Supplementary Fig.2. Analysis of the expression of Clics and siRNA-mediated knockdown effects in macrophages. (**a**) qPCR analysis of the expression of *Clics* in BMDMs with or without the treatment of LPS (50 ng ml^{-1}) for 6 h. Student's *t*-test. (**b**) Immunoblot analysis of Clics in BMDMs treated with LPS (50 ng ml^{-1}) at different time points. (**c**) Immunoblot analysis of Clics in LPS-primed BMDMs left stimulated with MSU at different time points. (**d, e**) qPCR (**d**) or immunoblot (**e**) analysis of the expression of *Clics* in BMDMs transfected with indicated siRNA against *Clics*. Data are from three independent experiments with biological duplicates in each (**a, d**; mean ± SEM of *n* = 6) or are representative of two independent experiments (**b, c, e**). *** P<0.001.

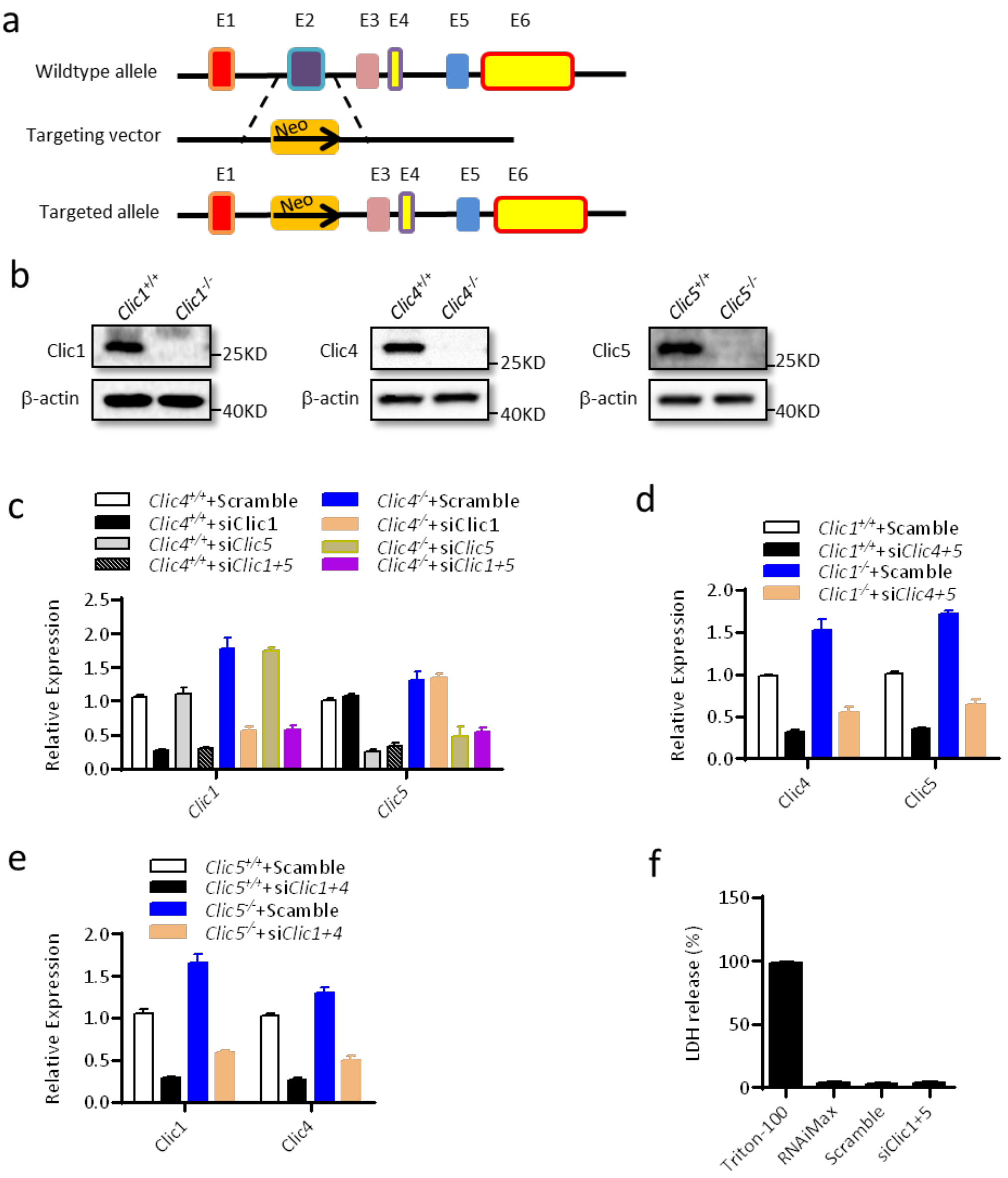

Supplementary Fig.3. Inhibition of *Clics* expression in macrophages. (**a**) *Clic4*$^{-/-}$ mice were generated by replacing exon 2 with a neomycin resistance cassette resulting in a truncated gene. (**b**) Immunoblot analysis of Clics in LPS-primed BMDMs from the indicated knockout mice lines. (**c-e**) qPCR analysis of the expression of *Clics* in *Clic4*$^{-/-}$ (**c**), *Clic1*$^{-/-}$ (**d**) or *Clic5*$^{-/-}$ (**e**) BMDMs transfected with the indicated siRNA. (**f**) Assay for LDH release in the culture supernatants of BMDMs transfected with the indicated siRNA. Data are from three independent experiments with biological duplicates in each (**c-f**; mean ± SEM of n = 6) or are representative of three independent experiments (**b**).

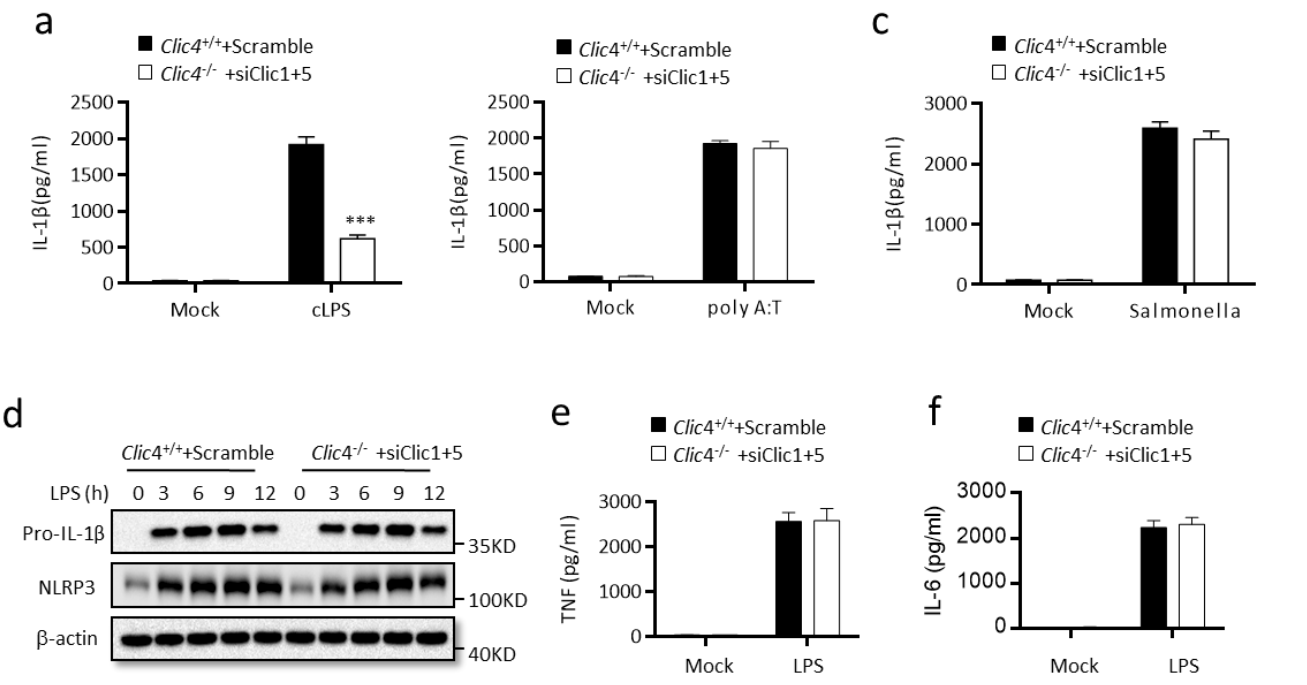

Supplementary Fig.4. Inhibition of Clics specifically inhibits NLRP3 inflammasome activation. (a-c) ELISA of IL-1β in culture supernatants of LPS-primed *Clic4*$^{-/-}$ BMDMs transfected with siRNA against *Clic1 and Clic5* and left stimulated with cytosolic LPS (cLPS) (**a**), poly A:T (**b**) or salmonella (**c**). Student's *t*-test (**a**). (**d**) Immunoblot analysis of indicated proteins in transfected with siRNA against *Clic1 and Clic5* and left stimulated with LPS for different time points. (**e, f**) ELISA of TNF (**e**) or IL-6 (**f**) in culture supernatants of transfected with siRNA against *Clic1 and Clic5* and left stimulated LPS for 6 hours. Data are from three independent experiments with biological duplicates in each (**a-c, e, f**; mean ± SEM of *n* = 6) or are representative of three independent experiments (**d**). *** P<0.001.

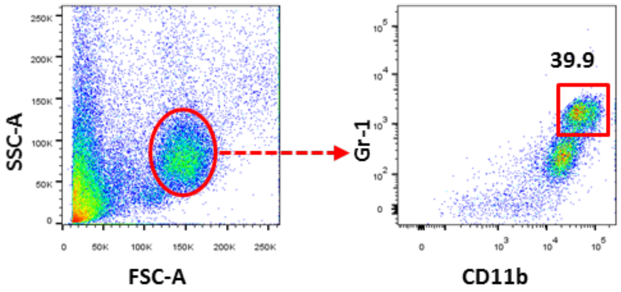

Supplementary Fig.5. Gate strategies of neutrophils in peritoneal lavage fluid. Peritoneal lavage fluid from C57BL/6 mice injected with MSU were assessed by flow cytometry. The Gated neutrophils are Gr-1^{+} CD11b^{hi}.

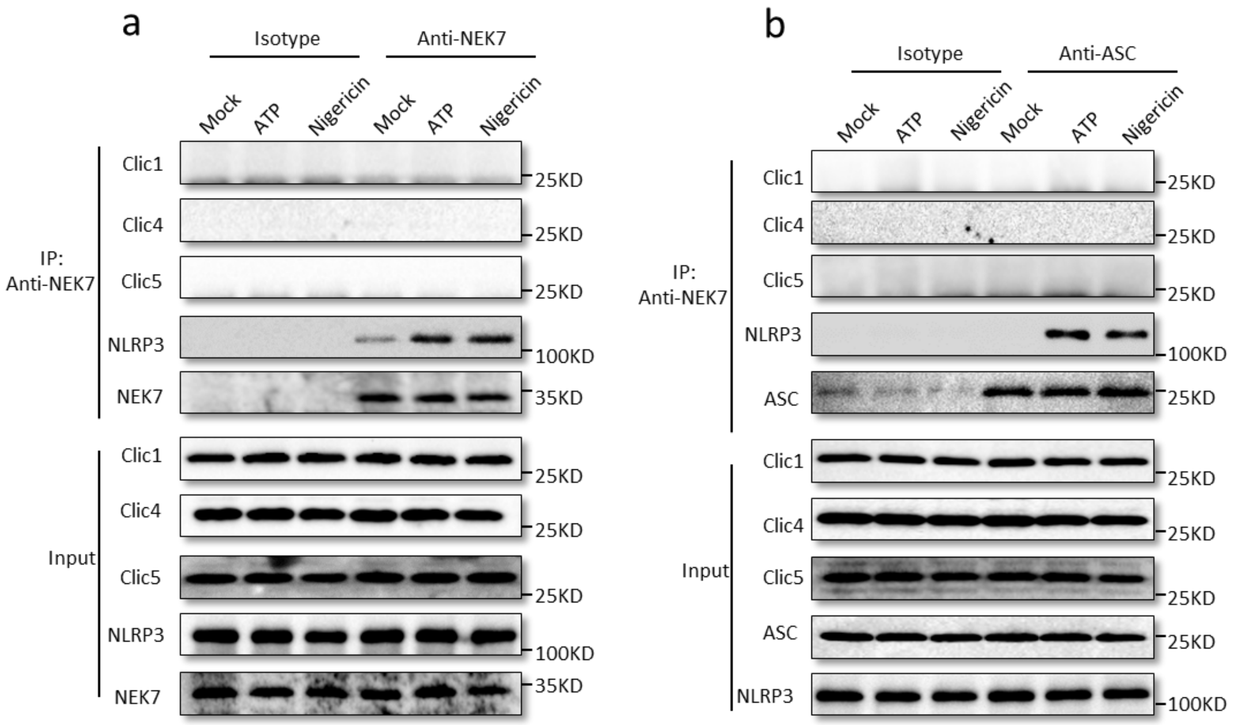

Supplementary Fig.6. Clics are not present in NLRP3 inflammasome complex during activation. (a) Immunoprecipitation (IP) and immunoblot analysis of the interaction of endogenous Clic1, 4, or 5 with NEK7-NLRP3 complex in LPS-primed BMDMs stimulated with ATP or nigericin. **(b)** Immunoprecipitation (IP) and immunoblot analysis of the interaction of endogenous Clic1, 4 or 5 with NLRP3-ASC complex in LPS-primed BMDMs stimulated with ATP or nigericin. Data are representative of two independent experiments (**a, b**).

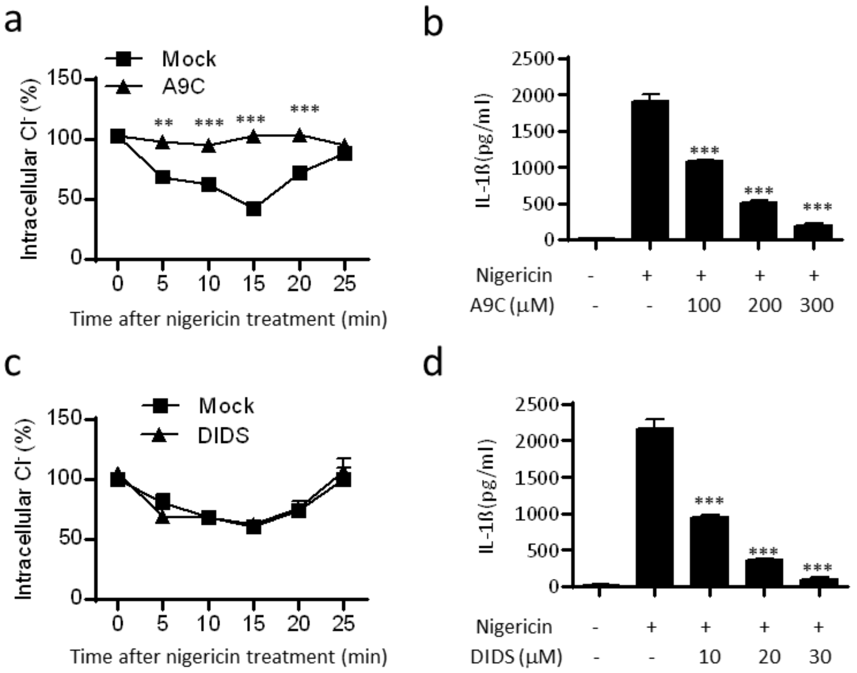

Supplementary Fig.7. A9C suppresses NLRP3 inflammasome activation and chloride efflux. (a) Qualification of the decrease of intracellular chloride in LPS-primed BMDMs at different time points after nigericin treatment with or without the pretreatment with A9C (300 μM). (**b**) ELISA analysis of IL-1β in culture supernatants of LPS-primed BMDMs treated with different doses of A9C and then left stimulated with nigericin. (**c**) Qualification of the decrease of intracellular chloride in LPS-primed BMDMs at different time points after nigericin treatment with or without the pretreatment with DIDS (30 μM). (**d**) ELISA analysis of IL-1β in culture supernatants of LPS-primed BMDMs treated with different doses of DIDS and then left stimulated with nigericin. Data are from three independent experiments with biological duplicates in each (**a-d**; mean ± SEM of $n = 6$). Two-way ANOVA (**b, d**), Student's *t*-test (**a**), ** $P<0.01$, *** $P<0.001$.

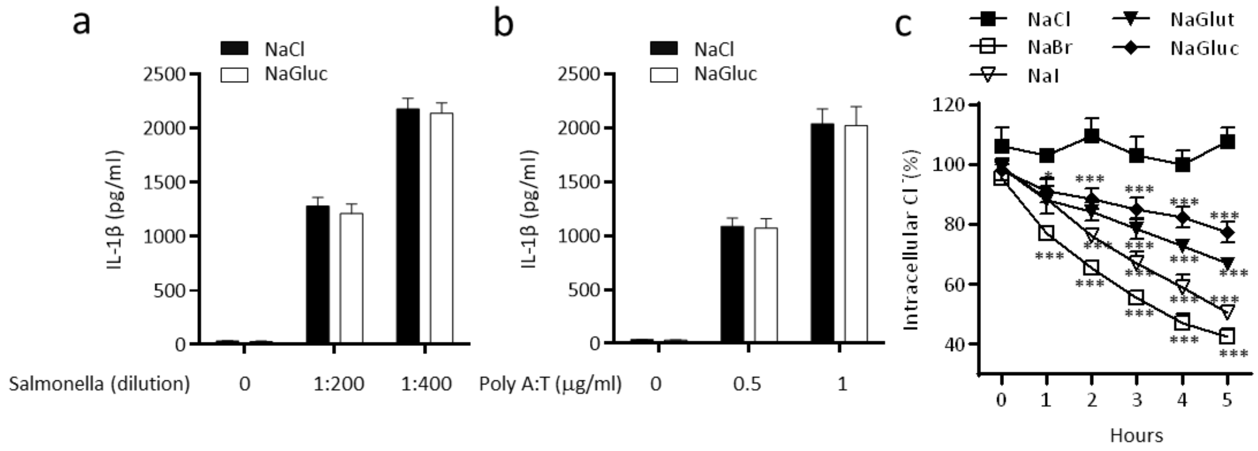

Supplementary Fig.8. Chloride efflux has no effect on AIM2 and NLRC4 inflammasome activation. (a, b) ELISA of IL-1β in LPS-primed BMDMs which were transferred to NaCl saline or chloride free NaGluc saline and left stimulated with salmonella (**a**) or poly A:T (**b**). (**c**) Qualification of the decrease of intracellular chloride in LPS-primed BMDMs which were transferred to NaCl, NaBr, NaI, NaGluc or NaGlut salines. Student's *t*-test. Data are from three independent experiments with biological duplicates in each (**a-c**; mean ± SEM of n = 6). ***P<0.001.

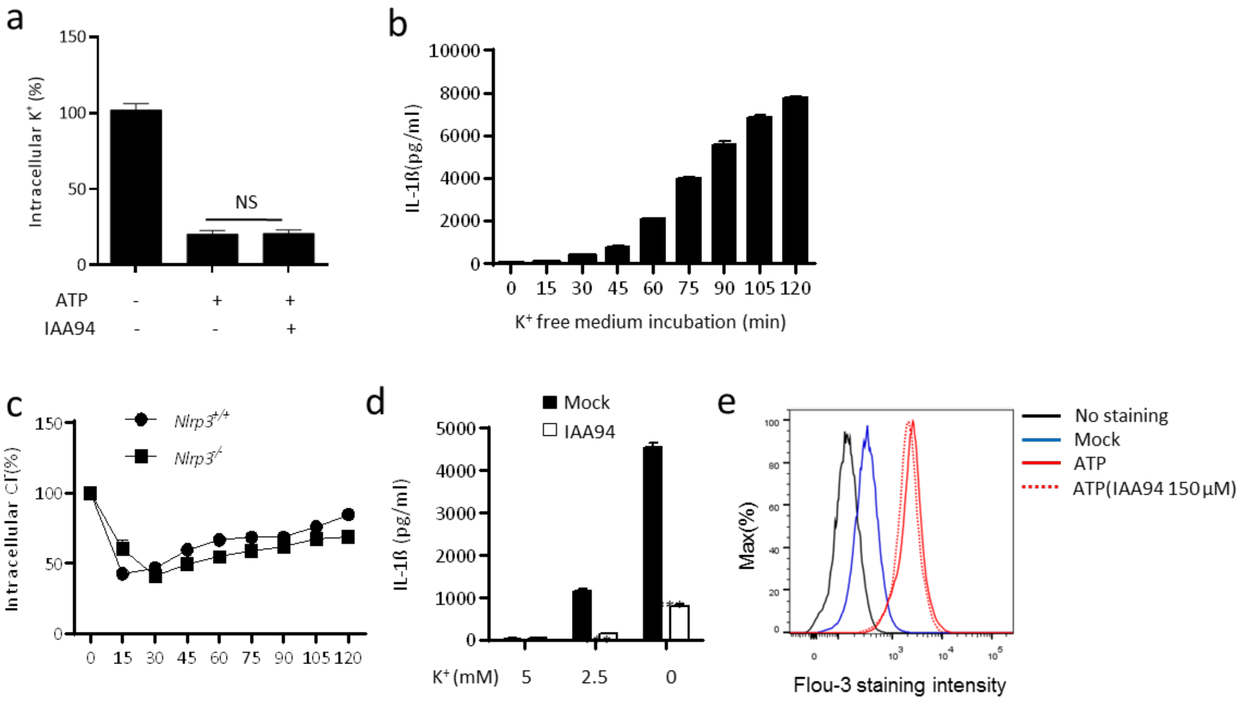

Supplementary Fig.9. Potassium free buffer-induced NLRP3 inflammasome depends on Clics. (a) Qualification of the decrease of intracellular potassium in LPS-primed BMDMs treated with IAA94 (150 μM) and then left stimulated with ATP. **(b)** ELISA of IL-1β in LPS-primed BMDMs which were transferred to potassium free saline for different time. (**c**) Qualification of the decrease of intracellular potassium in LPS-primed $Nlrp3^{+/+}$ or $Nlrp3^{-/-}$ BMDMs which were transferred to potassium free saline for different time. (**d**) ELISA of IL-1β in LPS-primed BMDMs which were transferred to potassium free saline for 3 h with or without the presence of IAA94 (150 μM). (**e**) Flow cytometry analysis of the intracellular calcium in LPS-primed BMDMs treated with IAA94 (150 μM) and then left stimulated with ATP. Data are from three independent experiments with biological duplicates in each (**a-d**, mean ± SEM of *n* = 6). NS, not significant, Student's *t*-test (**a, d**), *** P<0.001.

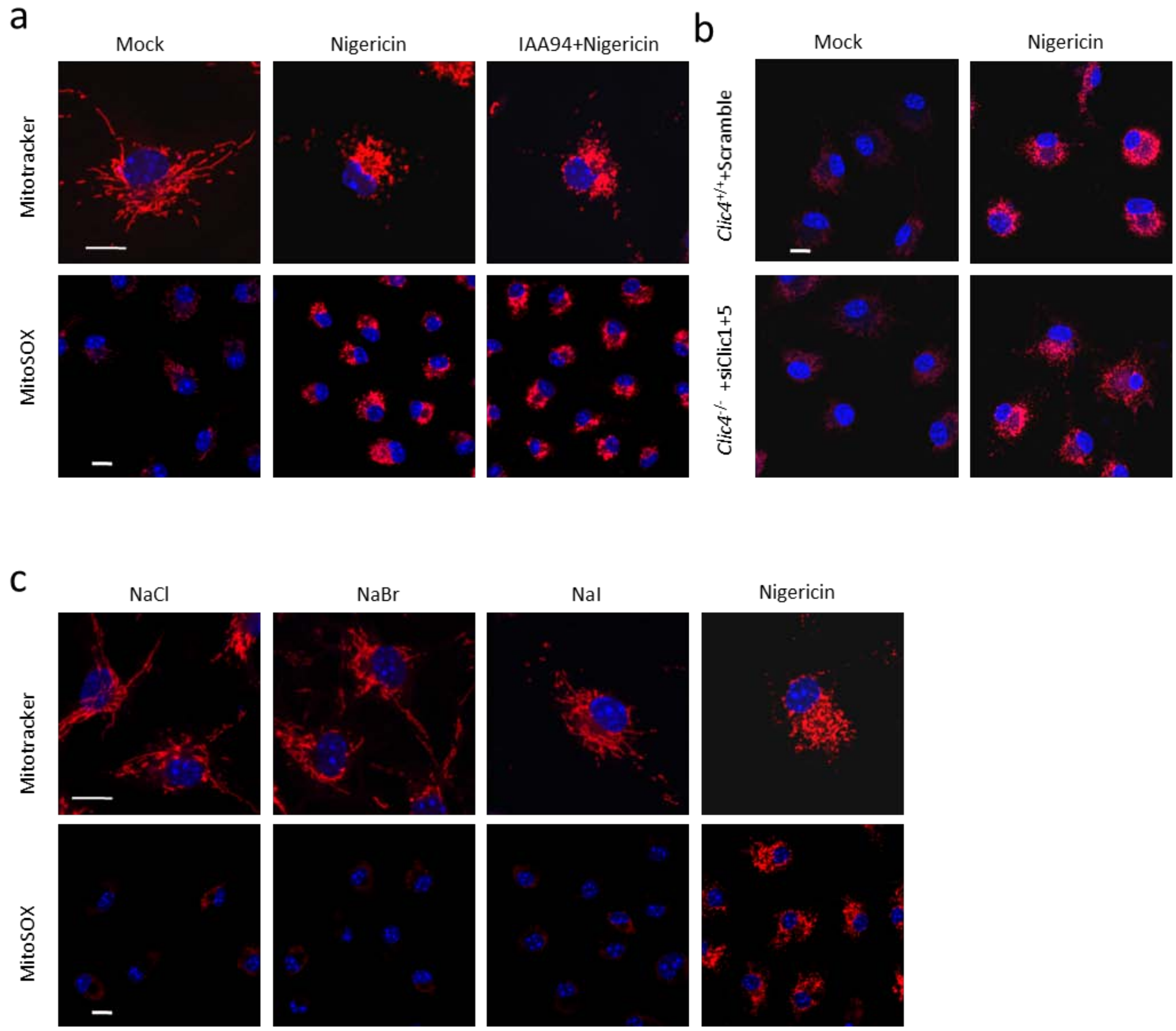

Supplementary Fig.10. Clics act downstream of mitochondrial ROS during NLRP3 activation. (a) Confocal microscopy analysis of LPS-primed BMDMs stimulated with nigericin with or without the presence of IAA94 (150 μM), followed by staining with Mitotracker red, Mitosox and DAPI. (**b**) Confocal microscopy analysis of LPS-primed *Clic4*$^{-/-}$ BMDMs transfected with siRNA against *Clic1* and *Clic5* and then left stimulated with nigericin, followed by staining with Mitosox red and DAPI. (**c**) Confocal microscopy analysis of LPS-primed BMDMs stimulated with nigericin for 30 min or transferred to NaCl, NaBr or NaI saline for 3 h, followed by staining with Mitotracker red, Mitosox and DAPI. Data are representative of three independent experiments. Scale bars, 10μm.

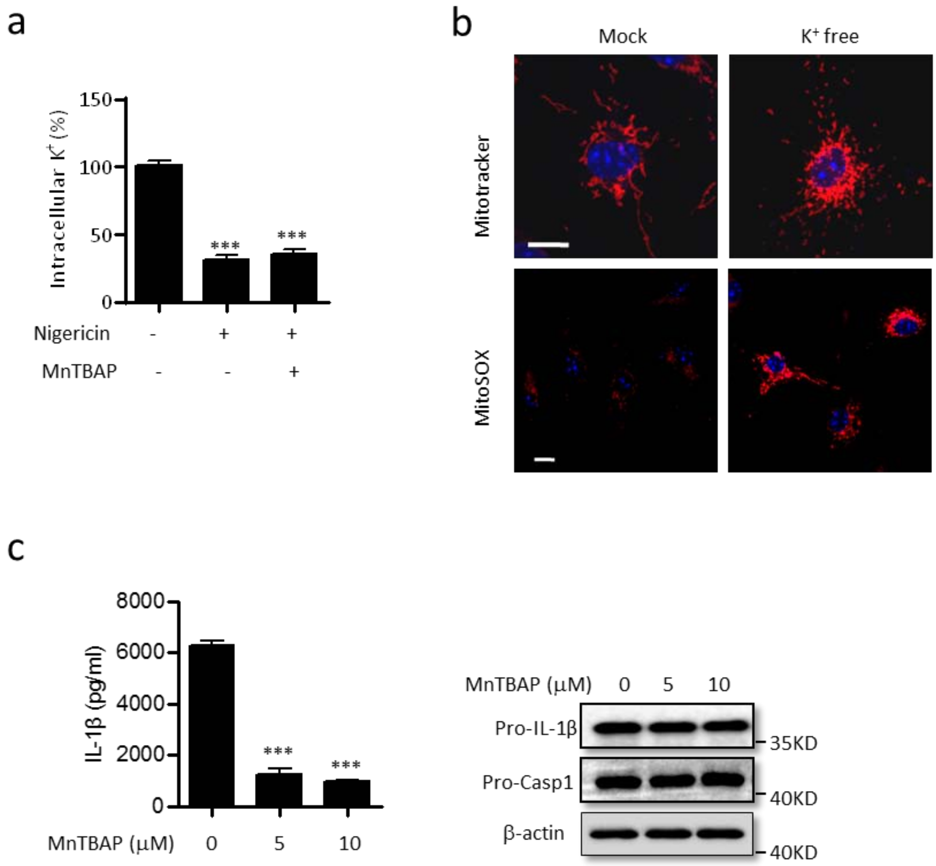

Supplementary Fig.11. Mitochondrial ROS acts downstream of potassium efflux during NLRP3 activation. **(a)** Qualification of the decrease of intracellular potassium in LPS-primed BMDMs stimulated with nigericin with or without the presence of MnTBAP (10 μM). (**b**) Confocal microscopy analysis of LPS-primed BMDMs transferred to potassium free saline for 3 h, followed by staining with Mitotracker red, Mitosox and DAPI. (**c**) ELISA of IL-1β or immunoblot analysis of indicated proteins in lysates of LPS-primed BMDMs which were transferred to potassium free saline for 3 hours with or without the presence of MnTBAP. Data are from three independent experiments with biological duplicates in each (**a, c**; mean ± SEM of n = 6) or are representative of three independent experiments (**b**). Two-way ANOVA (**a, c**), ***P<0.001.

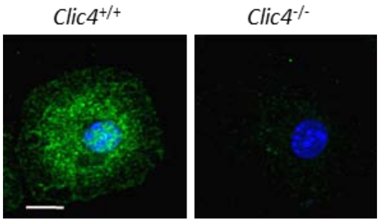

Supplementary Fig.12. Staining of endogenous Clic4 expression in BMDMs. Confocal microscopy analysis Clic4 in LPS-primed BMDMs from *Clic4*$^{+/+}$ or *Clic4*$^{-/-}$ mice. Data are representative of two independent experiments.

Supplementary Fig. 13. Scans of the full films used to generate Western blot data for figure 1a-2d.

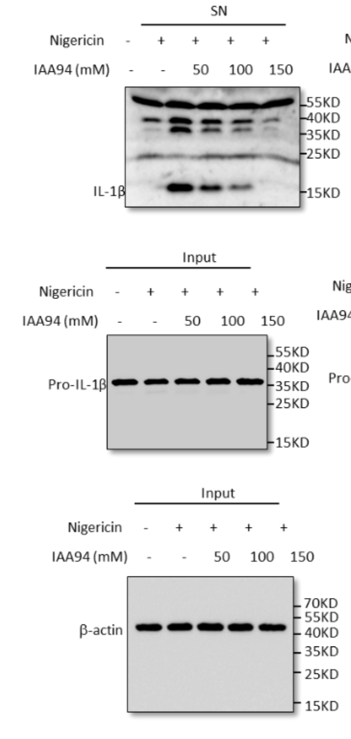

Fig.1a

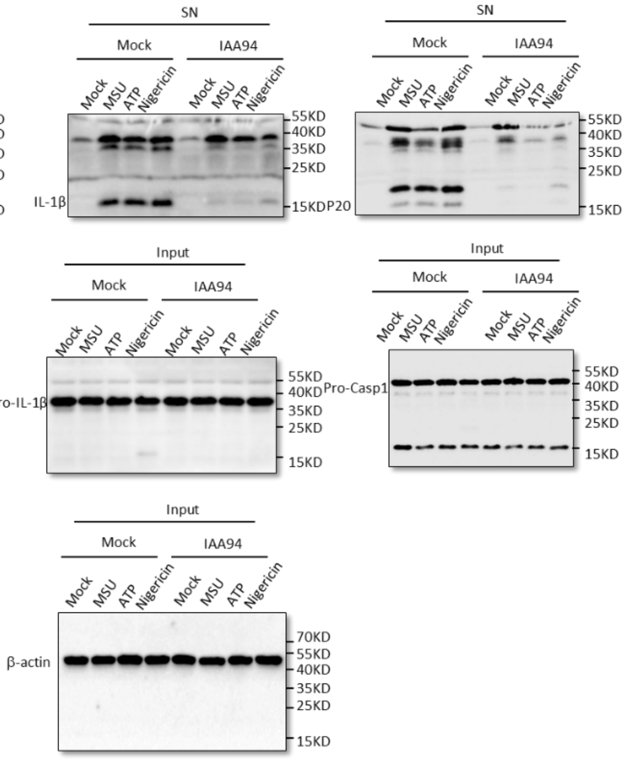

Fig.1d

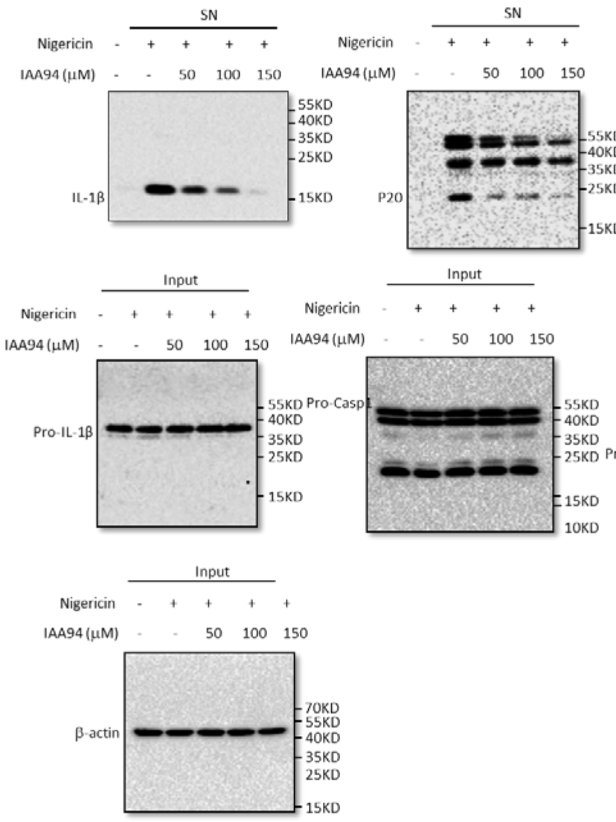

Fig.1f

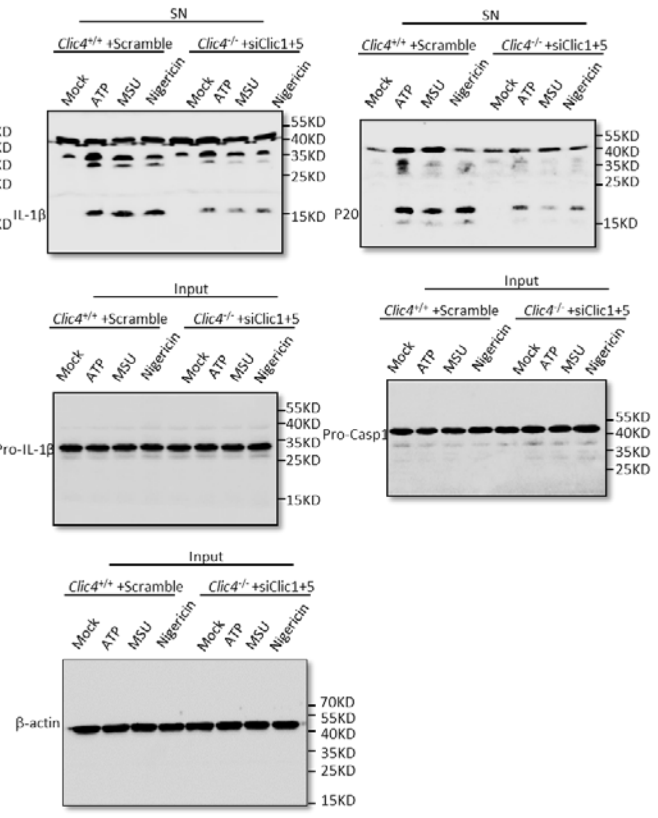

Fig.2d

Supplementary Fig.14. Scans of the full films used to generate Western blot data for figure 5e-7b.

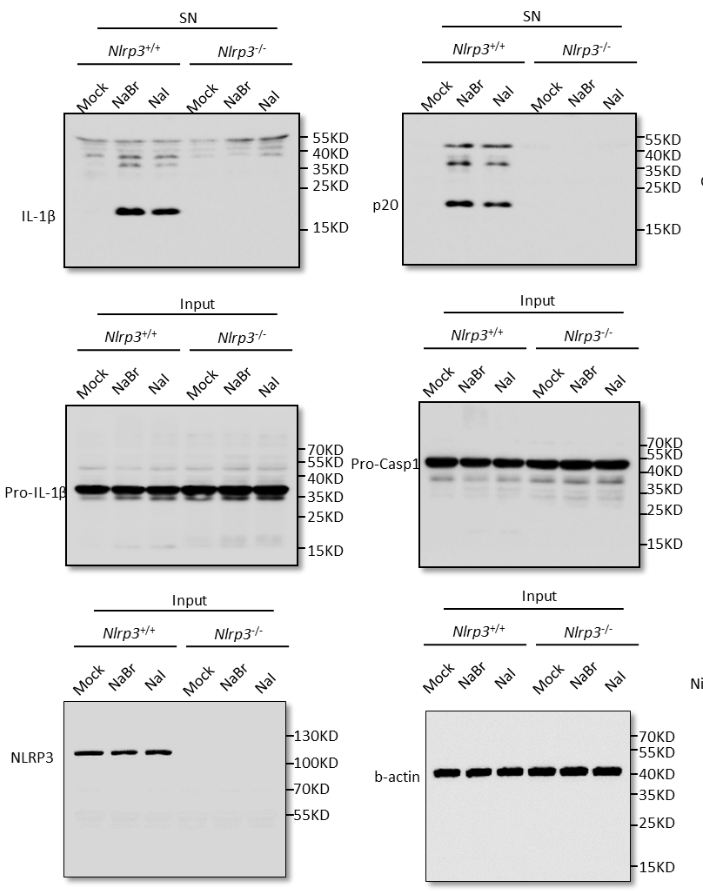

Fig.5e

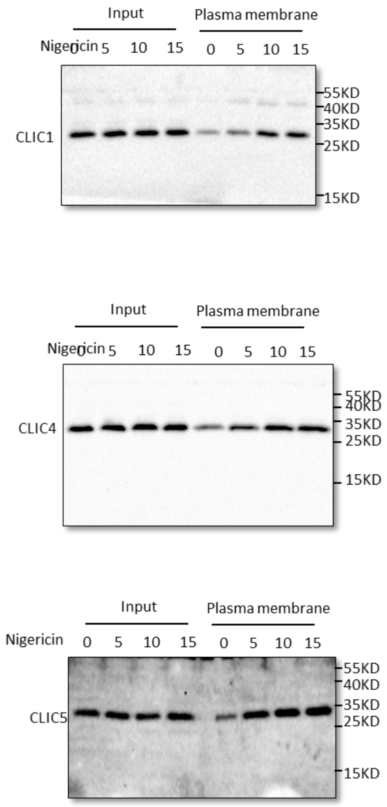

Fig.7a

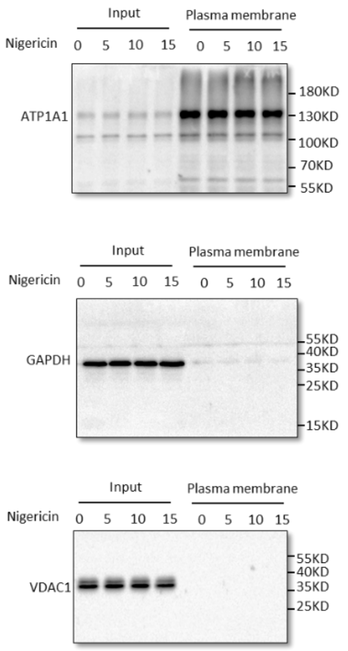

Fig.7a

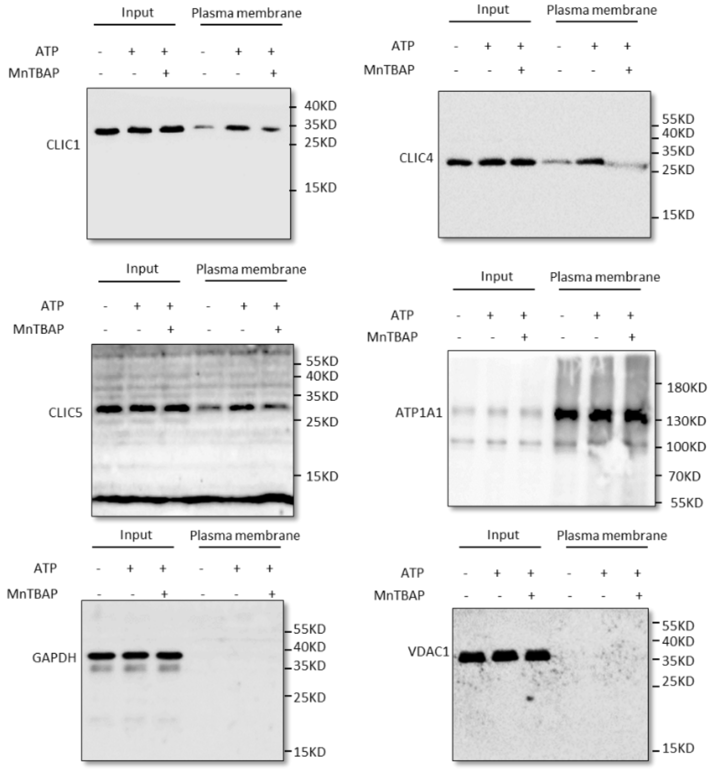

Fig.7b

Supplementary Fig.15. Scans of the full films used to generate Western blot data for figure 7c-8d.

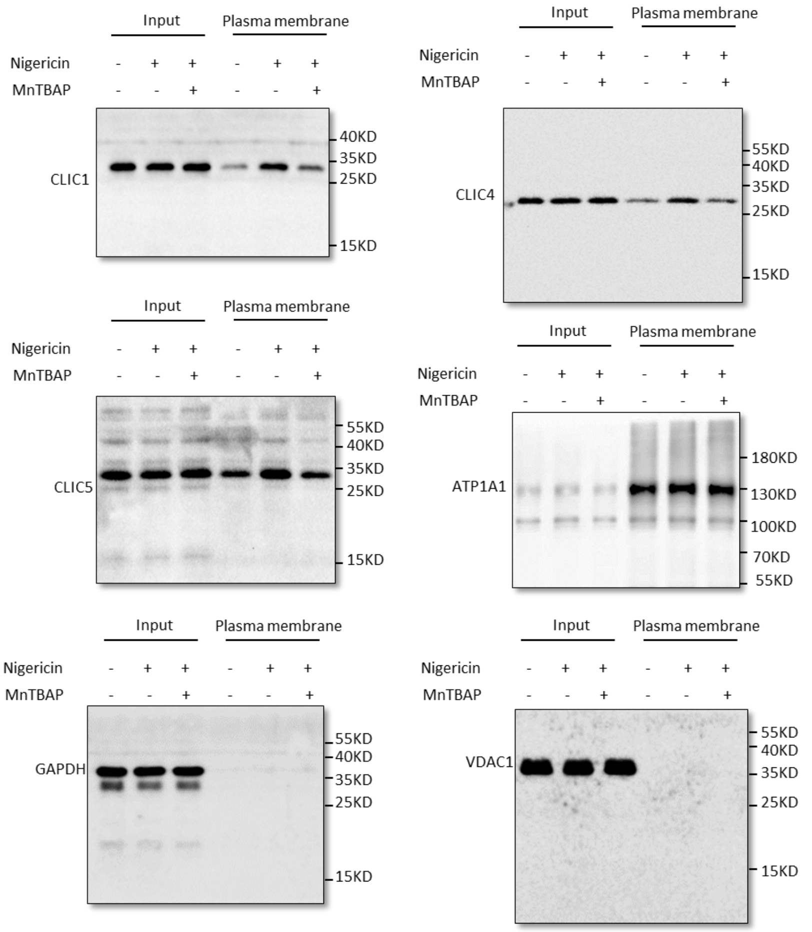

Fig.7c

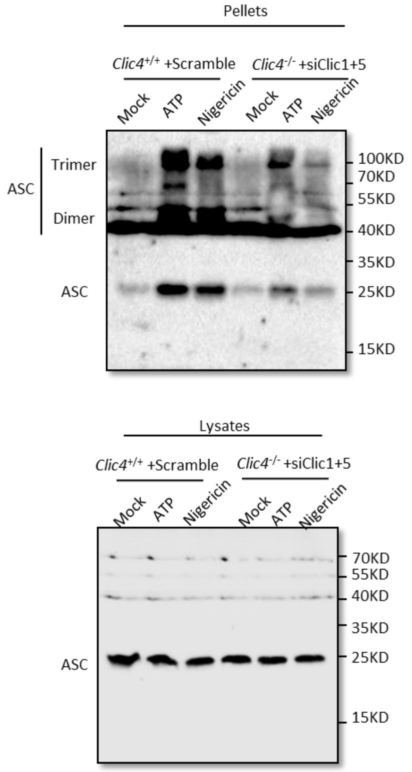

Fig.8a

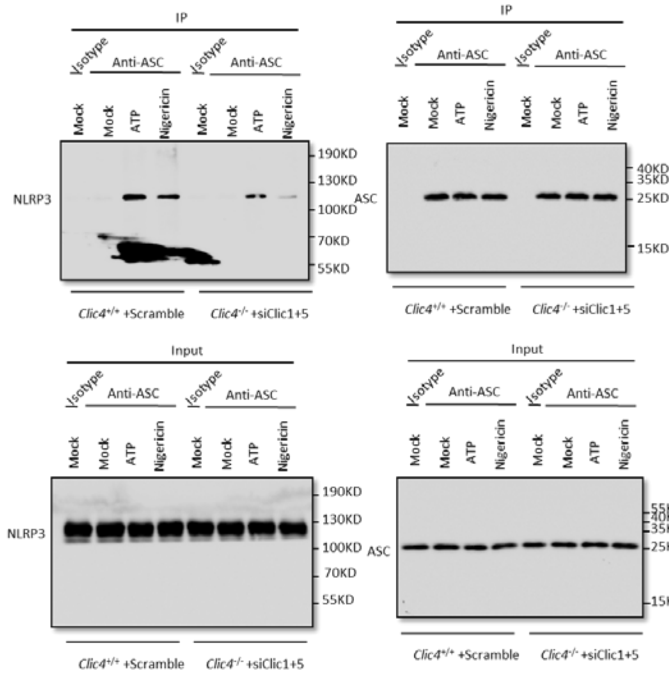

Fig.8b

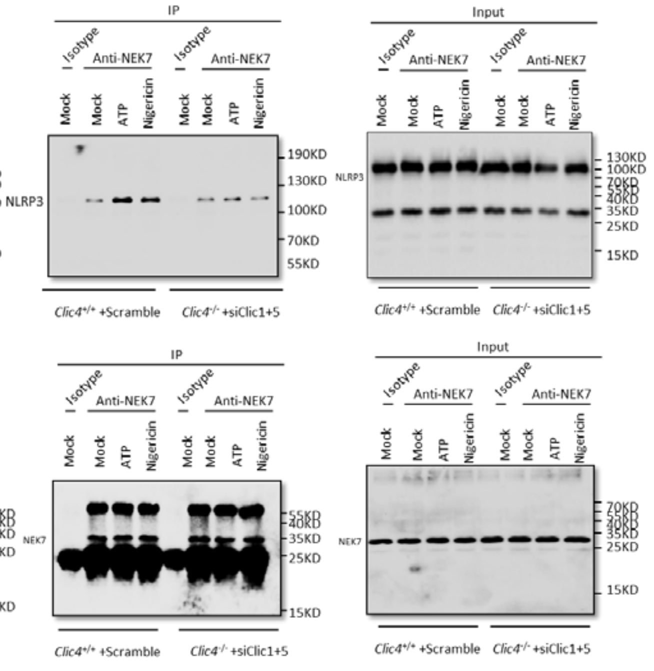

Fig.8d

Supplementary Fig.16. Scans of the full films used to generate Western blot data for supplementary figure 1a-2e.

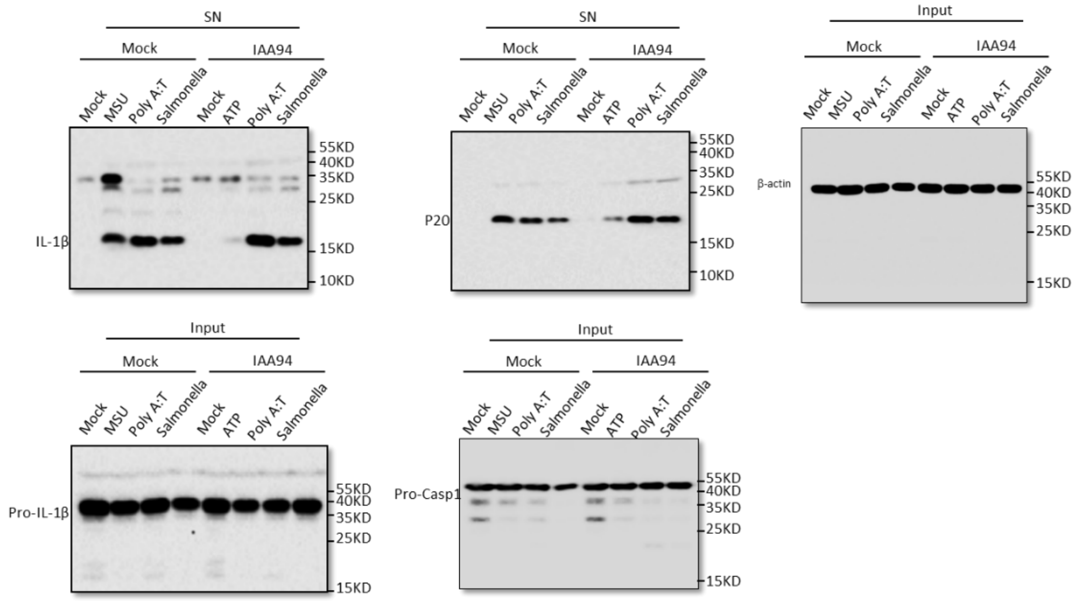

Supplementary Fig. 1a

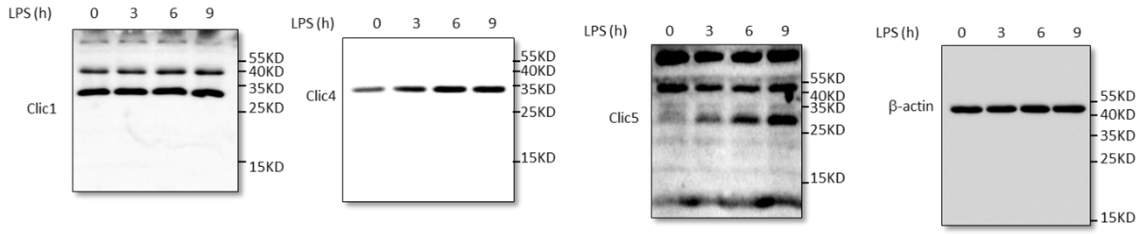

Supplementary Fig. 2b

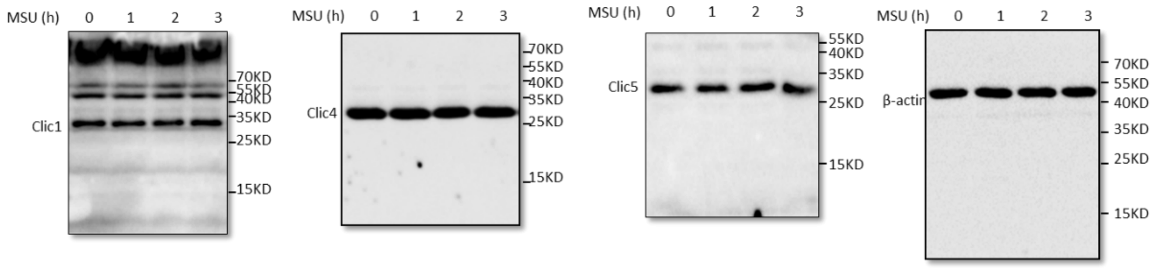

Supplementary Fig. 2c

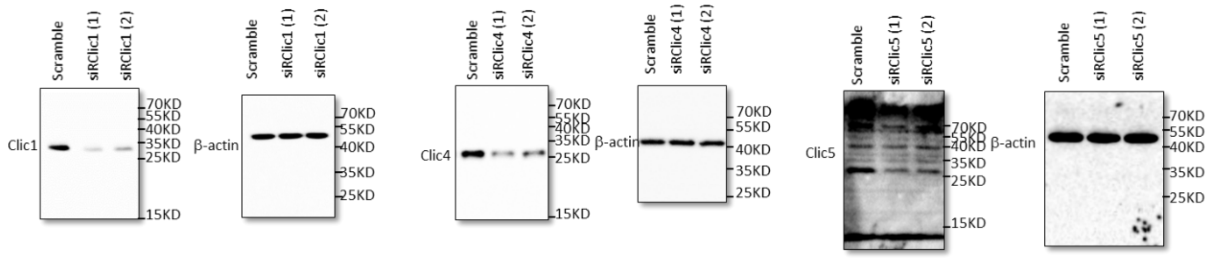

Supplementary Fig. 2e

Supplementary Fig.17. Scans of the full films used to generate Western blot data for supplementary figure 3b-6a.

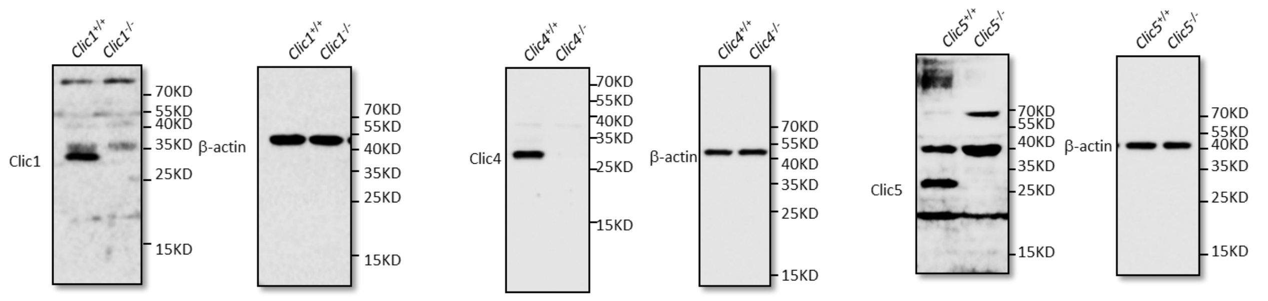

Supplementary Fig. 3b

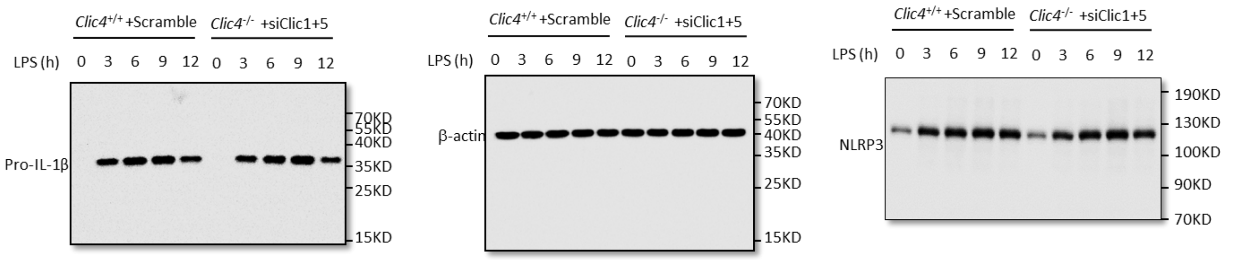

Supplementary Fig. 4d

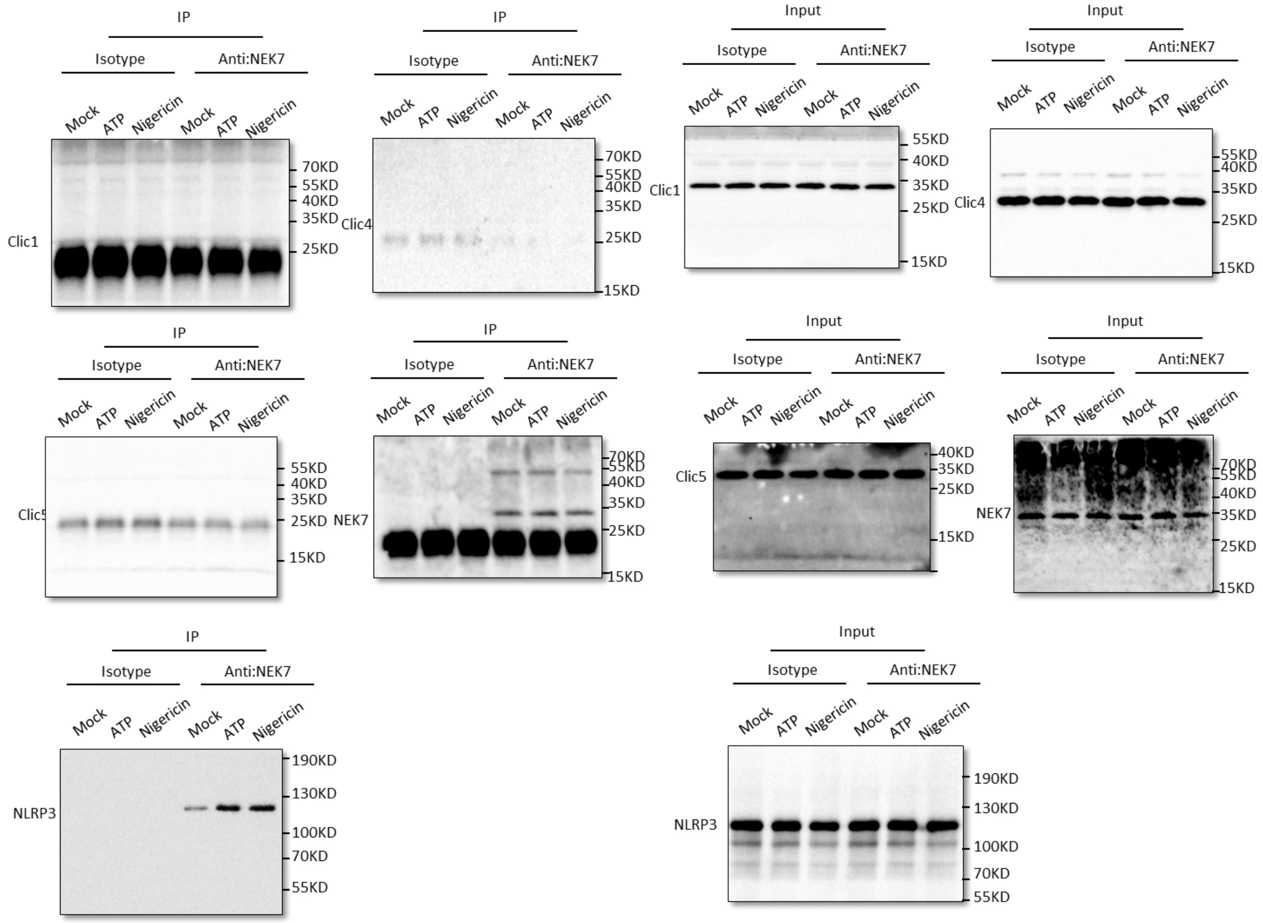

Supplementary Fig. 6a

Supplementary Fig.18. Scans of the full films used to generate Western blot data for supplementary figure 6b-11d.

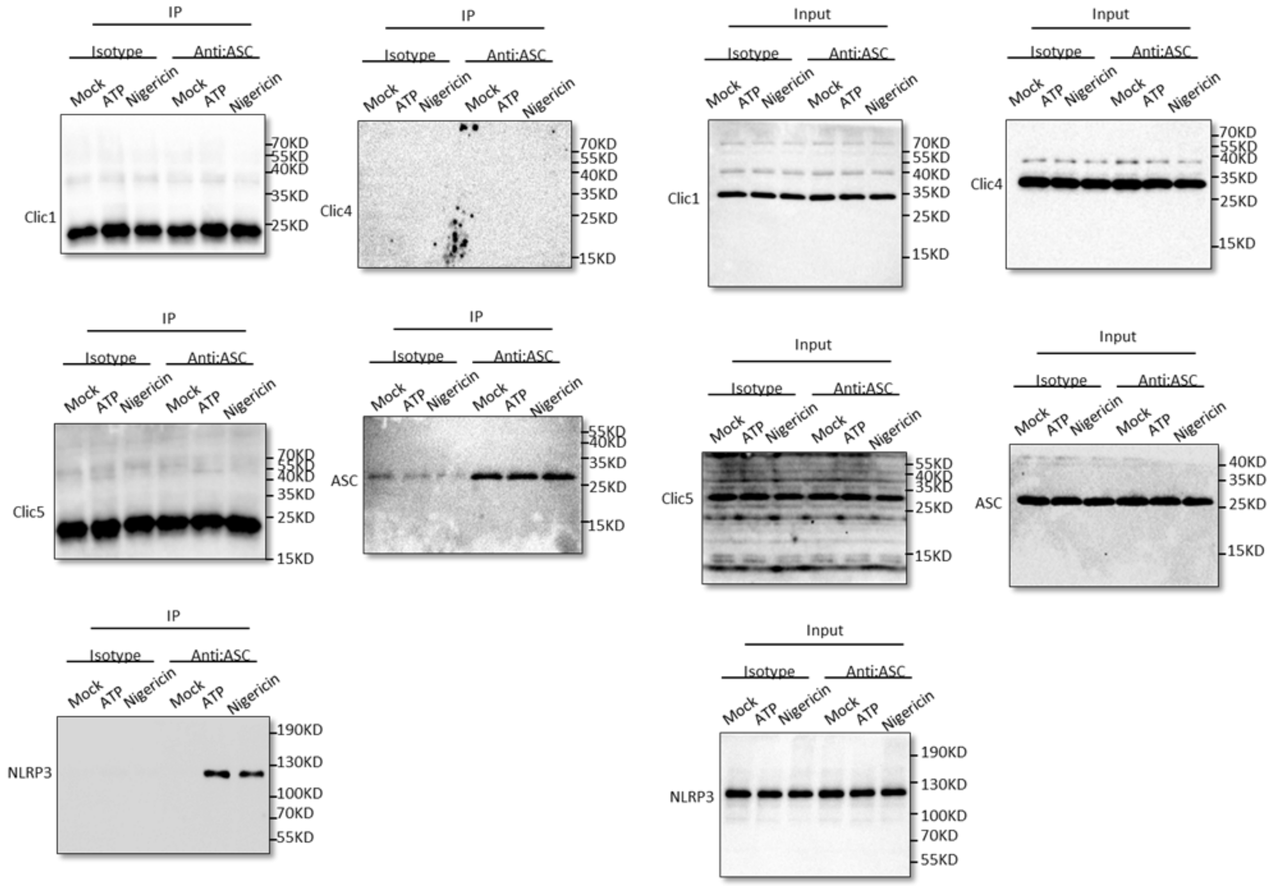

Supplementary Fig. 6b

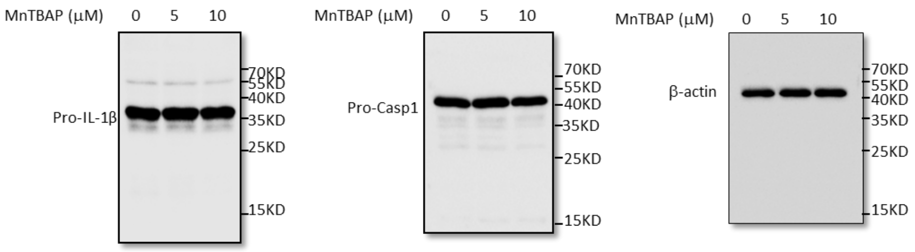

Supplementary Fig. 11d

Supplementary Table 1. siRNAs used in the paper

siRNA	Sequences
Scramble	5'-TTCTCCGAACGTGTCACGT-3'
siClic1(1)	5'- CACCAACAAGATCGAGGAATT -3'
siClic1(2)	5'- GCCCTGAAGGTTCTAGACAAT -3'
siClic4(1)	5'- GCTTACATCAAGAACTCAAGA -3'
siClic4(2)	5'- GAAGTGATGGTGAAAGCATTG -3'
siClic5(1)	5'- GCTAAGAAGTACCGAAACTAT -3'
siClic5(2)	5'- CGATACCTCAAGAATGCCTAT -3'

Supplementary Table 2. qRT-PCR primers used in the paper

Gene name	GeneBank Accession	Sequence of forword primer	Sequence of reverse primer
Clic1	NM_033444.2	5'-ctcccaggtacccaaagctg-3'	5'-tgccgtccagaaacttcctc-3'
Clic4	NM_013885.2	5'- gctttcaccaaagcacccag-3'	5'- atgtcatctcatcgccgtcc-3'
Clic5	NM_172621.2	5'- ctggctgaaaggagtcgtgt-3'	5'- gtttggggtacttctcgggg-3'
GAPDH	NM_001115114.1	5'-ggtgaaggtcggtgtgaacg-3'	5'-ctcgctcctggaagatggtg-3'
